# Supplementary material for: Genetic background of high myopia in children
Source: PLoS One. 2024 Nov 4;19(11):e0313121. doi: 10.1371/journal.pone.0313121 (PMC11534203; doi:10.1371/journal.pone.0313121)
Supplement: S1 Table — (DOCX) [file pone.0313121.s001.docx]

**S1 Table. Eye disorder gene panel.**

| ABCA4 |
| --- |
| ABCB6 |
| ABCC6 |
| ABHD12 |
| ACBD5 |
| ACO2 |
| ACTB |
| ACTG1 |
| ADAM9 |
| ADAMTS10 |
| ADAMTS17 |
| ADAMTS18 |
| ADAMTS2 |
| ADAMTSL4 |
| ADGRV1 |
| ADIPOR1 |
| AFG3L2 |
| AGBL1 |
| AGBL5 |
| AGK |
| AHI1 |
| AHR |
| AIPL1 |
| ALDH18A1 |
| ALDH1A3 |
| ALMS1 |
| ALX3 |
| ANTXR1 |
| AP3B1 |
| AP3D1 |
| ARHGAP31 |
| ARHGEF18 |
| ARL13B |
| ARL2 |
| ARL2BP |
| ARL3 |
| ARL6 |
| ARMC9 |
| ARR3 |
| ARSG |
| ASB10 |
| ASPH |
| ASRGL1 |
| ASXL1 |
| ATF6 |
| ATOH7 |
| ATP1A3 |
| B3GALT6 |
| B3GLCT |
| B9D1 |
| B9D2 |
| BBIP1 |
| BBS1 |
| BBS10 |
| BBS12 |
| BBS2 |
| BBS4 |
| BBS5 |
| BBS7 |
| BBS9 |
| BCOR |
| BEST1 |
| BFSP1 |
| BFSP2 |
| BLOC1S3 |
| BLOC1S6 |
| BMP4 |
| BMP7 |
| BSG |
| C12orf57 |
| C19ORF12 |
| C1QTNF5 |
| C3 |
| C8ORF37 |
| CA4 |
| CABP4 |
| CACNA1F |
| CACNA2D4 |
| CAPN5 |
| CARS1 |
| CBS |
| CC2D2A |
| CCT2 |
| CDH2 |
| CDH23 |
| CDH3 |
| CDHR1 |
| CEP104 |
| CEP120 |
| CEP164 |
| CEP250 |
| CEP290 |
| CEP41 |
| CEP78 |
| CEP83 |
| CERKL |
| CFAP410 |
| CFAP418 |
| CFB |
| CFH |
| CFI |
| CHD7 |
| CHM |
| CHMP4B |
| CHRDL1 |
| CHST14 |
| CHST6 |
| CIB2 |
| CISD2 |
| CLCC1 |
| CLDN19 |
| CLN3 |
| CLN5 |
| CLN6 |
| CLN8 |
| CLRN1 |
| CLUAP1 |
| CNGA1 |
| CNGA3 |
| CNGB1 |
| CNGB3 |
| CNNM4 |
| COL11A1 |
| COL11A2 |
| COL18A1 |
| COL25A1 |
| COL2A1 |
| COL4A1 |
| COL5A1 |
| COL8A2 |
| COL9A1 |
| COL9A2 |
| COL9A3 |
| CPAMD8 |
| CPLANE1 |
| CPSF1 |
| CRB1 |
| CRX |
| CRYAA |
| CRYAB |
| CRYBA1 |
| CRYBA2 |
| CRYBA4 |
| CRYBB1 |
| CRYBB2 |
| CRYBB3 |
| CRYGB |
| CRYGC |
| CRYGD |
| CRYGS |
| CSPP1 |
| CTDP1 |
| CTNNA1 |
| CTNNB1 |
| CTNND1 |
| CTSD |
| CTSH |
| CWC27 |
| CYP1B1 |
| CYP4V2 |
| DCN |
| DHDDS |
| DHX38 |
| DKC1 |
| DNM1L |
| DNMBP |
| DOCK6 |
| DPYD |
| DRAM2 |
| DTHD1 |
| DTNBP1 |
| DZIP1 |
| EFEMP1 |
| ELOVL1 |
| ELOVL4 |
| ELP4 |
| EMC1 |
| EPG5 |
| EPHA2 |
| EPHB2 |
| ERBB3 |
| EXOSC2 |
| EYA1 |
| EYS |
| FA2H |
| FAM161A |
| FBN1 |
| FBN2 |
| FGFR3 |
| FKBP14 |
| FLVCR1 |
| FOXC1 |
| FOXE3 |
| FOXL2 |
| FRAS1 |
| FREM1 |
| FREM2 |
| FRMD7 |
| FSCN2 |
| FTL |
| FYCO1 |
| FZD4 |
| GALK1 |
| GALM |
| GALT |
| GCNT2 |
| GDF3 |
| GDF6 |
| GFER |
| GJA1 |
| GJA3 |
| GJA8 |
| GLI2 |
| GLIS2 |
| GNAT1 |
| GNAT2 |
| GNB3 |
| GNPTG |
| GPR143 |
| GPR179 |
| GRASP |
| GRHL2 |
| GRIP1 |
| GRK1 |
| GRM6 |
| GRN |
| GSN |
| GUCA1A |
| GUCA1B |
| GUCY2D |
| GZF1 |
| HARS1 |
| HCCS |
| HGSNAT |
| HK1 |
| HMGB3 |
| HMX1 |
| HPS1 |
| HPS3 |
| HPS4 |
| HPS5 |
| HPS6 |
| HS6ST2 |
| HSF4 |
| IDH3A |
| IDH3B |
| IFIH1 |
| IFT140 |
| IFT172 |
| IFT27 |
| IFT43 |
| IFT52 |
| IFT74 |
| IFT81 |
| IGBP1 |
| IMPDH1 |
| IMPG1 |
| IMPG2 |
| INPP5E |
| INVS |
| IPO13 |
| IQCB1 |
| IRX5 |
| JAG1 |
| JAM3 |
| KCNJ13 |
| KCNV2 |
| KERA |
| KIAA0556 |
| KIAA0586 |
| KIAA1549 |
| KIF11 |
| KIF21A |
| KIF3B |
| KIF7 |
| KIZ |
| KLHL7 |
| KRT12 |
| KRT3 |
| LAMA1 |
| LAMB2 |
| LCA5 |
| LCAT |
| LEMD2 |
| LIM2 |
| LOXL3 |
| LRAT |
| LRIT3 |
| LRMDA |
| LRP2 |
| LRP5 |
| LRPAP1 |
| LRRC32 |
| LSS |
| LTBP2 |
| LYST |
| LZTFL1 |
| MAB21L2 |
| MAF |
| MAK |
| MAPKAPK3 |
| MERTK |
| MFN2 |
| MFRP |
| MFSD8 |
| MIP |
| MIR184 |
| MIR204 |
| MITF |
| MKKS |
| MKS1 |
| MLPH |
| MMADHC |
| MTTP |
| MVK |
| MYCBP2 |
| MYO5A |
| MYO7A |
| MYOC |
| NAA10 |
| NAGLU |
| NBAS |
| NDP |
| NDUFAF7 |
| NEK2 |
| NEK8 |
| NEUROD1 |
| NHS |
| NIPBL |
| NMNAT1 |
| NPHP1 |
| NPHP3 |
| NPHP4 |
| NR2E3 |
| NR2F1 |
| NRL |
| NYX |
| OAT |
| OCA2 |
| OCRL |
| OFD1 |
| OPA1 |
| OPA3 |
| OPN1LW |
| OPN1MW |
| OPN1SW |
| OPTN |
| OR2W3 |
| OTX2 |
| OVOL2 |
| P3H2 |
| P4HA1 |
| P4HA2 |
| PACS1 |
| PAK2 |
| PANK2 |
| PAX2 |
| PAX6 |
| PCARE |
| PCDH15 |
| PCYT1A |
| PDE6A |
| PDE6B |
| PDE6C |
| PDE6D |
| PDE6G |
| PDE6H |
| PDZD7 |
| PEX1 |
| PEX2 |
| PEX26 |
| PEX6 |
| PEX7 |
| PGK1 |
| PHOX2A |
| PHYH |
| PIGL |
| PIK3R1 |
| PIKFYVE |
| PITPNM3 |
| PITX2 |
| PITX3 |
| PLA2G5 |
| PLK4 |
| PLOD1 |
| PLOD3 |
| PNPLA6 |
| POC1B |
| POC5 |
| POLR3B |
| POMGNT1 |
| POMT1 |
| PPT1 |
| PRCD |
| PRDM13 |
| PRDM5 |
| PRIMPOL |
| PROM1 |
| PRPF3 |
| PRPF31 |
| PRPF4 |
| PRPF6 |
| PRPF8 |
| PRPH2 |
| PRSS56 |
| PXDN |
| RAB28 |
| RAB3GAP2 |
| RARB |
| RAX |
| RAX2 |
| RBP3 |
| RBP4 |
| RCBTB1 |
| RD3 |
| RDH11 |
| RDH12 |
| RDH5 |
| REEP6 |
| RGR |
| RGS9 |
| RGS9BP |
| RHO |
| RIMS1 |
| RIMS2 |
| RLBP1 |
| ROM1 |
| RP1 |
| RP1L1 |
| RP2 |
| RP9 |
| RPE65 |
| RPGR |
| RPGRIP1 |
| RPGRIP1L |
| RS1 |
| RTN4IP1 |
| SAG |
| SALL2 |
| SALL4 |
| SAMD11 |
| SC5D |
| SCAPER |
| SCO2 |
| SDCCAG8 |
| SEMA4A |
| SGSH |
| SHH |
| SIPA1L3 |
| SIX3 |
| SIX6 |
| SLC16A12 |
| SLC24A1 |
| SLC24A5 |
| SLC25A46 |
| SLC33A1 |
| SLC38A8 |
| SLC39A5 |
| SLC45A2 |
| SLC4A11 |
| SLC52A2 |
| SLC7A14 |
| SLITRK6 |
| SMOC1 |
| SMS |
| SNRNP200 |
| SOX2 |
| SOX5 |
| SPATA7 |
| SPP2 |
| SRD5A3 |
| SSBP1 |
| STRA6 |
| SUFU |
| TACSTD2 |
| TBC1D24 |
| TCF4 |
| TCOF1 |
| TCTN1 |
| TCTN2 |
| TCTN3 |
| TDRD7 |
| TEAD1 |
| TEK |
| TENM3 |
| TFAP2A |
| TGFBI |
| TGFBR1 |
| TGFBR2 |
| TGIF1 |
| TIMM8A |
| TIMP3 |
| TMEM107 |
| TMEM126A |
| TMEM138 |
| TMEM216 |
| TMEM231 |
| TMEM237 |
| TMEM67 |
| TMEM98 |
| TNFRSF21 |
| TOPORS |
| TPP1 |
| TRAF3IP1 |
| TREX1 |
| TRIM32 |
| TRNT1 |
| TRPM1 |
| TSPAN12 |
| TTC8 |
| TTLL5 |
| TUB |
| TUBA3D |
| TUBB3 |
| TUBB4B |
| TUBGCP4 |
| TUBGCP6 |
| TULP1 |
| TYR |
| TYRP1 |
| UBIAD1 |
| UNC119 |
| UNC45B |
| USH1C |
| USH1G |
| USH2A |
| USP45 |
| VAX1 |
| VCAN |
| VIM |
| VPS13B |
| VSX1 |
| VSX2 |
| WASHC5 |
| WDPCP |
| WDR19 |
| WDR36 |
| WFS1 |
| WHRN |
| WRN |
| XYLT1 |
| YAP1 |
| YME1L1 |
| ZEB1 |
| ZNF408 |
| ZNF423 |
| ZNF469 |
| ZNF513 |
| ZNF644 |
