# Supplementary material for: Genetic background of high myopia in children
Source: PLoS One. 2024 Nov 4;19(11):e0313121. doi: 10.1371/journal.pone.0313121 (PMC11534203; doi:10.1371/journal.pone.0313121)
Supplement: Attachment — Submitted filename: Cover letter 2.docx [file pone.0313121.s002.docx]

PONE-D-24-12970

***Manuscript title:*** Genetic background of high myopia in children

Dear Ms Irene Nathalie Fernandez Tolentino,

Thank you very much for the assessment of our submitted manuscript regarding genetics of high myopia in children. We sincerely appreciate the time, effort and feedback on our manuscript to improve the quality of the manuscript.

We have been through each of the comments and addressed/made changes accordingly. This is detailed below by our comments in blue italic text to each of the comments raised. Revised manuscript file has been uploaded electronically.

(i) You have reported that the data were accessed between ‘22/12/2022’ and ‘17/01/2022’ for Clinical Institute of Genomic Medicine (CIGM) . There appears to be a timeline error. Kindly provide the correct dates.

*Thank you for spotting this error. The correct dates were provided.*

(ii)Kindly upload a ‘single updated Manuscript’ with the correct date of data access.

*A single updated Manuscript with correct date of data access was uploaded as suggested.*

(iii) Upload a copy of the original ethics approval letter and also provide an English translated version as a supporting file separately in pdf format.

*Both, the original ethics approval letter in Slovene language as well as an English translated version were provided as a supporting file in pdf.*

(iv) Clarify the discrepancy in the number of participants. In the IRB approval it states that 30 subjects will be enrolled, but figure it is stated as 37.

*We had indicated on the application form for the Republic of Slovenia National Medical Ethics Committee that we intended to include 30 children in our study. Our study design had estimated that around 30 children undergoing treatment at the eye clinic in the indicated period would meet the inclusion criteria and agree to undergo genetic testing, and their results would be used for our analysis. However, during our survey, we found more than 30 such children and we decided to include all of them in our study, as we felt it would not be appropriate to exclude some of them based solely on the estimation, we made during the study design phase. It should be mentioned that the total number of 46 children, stated in our manuscript, also includes those who subsequently did not agree to genetic testing or were unavailable and were therefore not included in the final analysis. The analysis was performed on a total of 36 probands (37 children, two of whom were siblings).*

We hope our comments are satisfactory but of course are happy to consider any additional points should there be any.

We look forward to hearing from you.
With kindest regards,

**Dr Manca Tekavcic Pompe** *On behalf of all co-authors*
Emal: [manca.tekavcic-pompe@guest.arnes.si](mailto:manca.tekavcic-pompe@guest.arnes.si)
